# Both/And: Mixed methods analysis of network composition, communication patterns, and socio-economic support within social networks of transgender women in Lima, Peru

**DOI:** 10.21203/rs.3.rs-3150364/v1

**Published:** 2023-07-31

**Authors:** Tijana Temelkovska, Kathleen Moriarty, Leyla Huerta, Amaya G. Perez-Brumer, Eddy R. Segura, Ryan Colby Passaro, Jordan E. Lake, Jesse L. Clark, Cheríe S. Blair

**Affiliations:** University of Colorado; MedStar Georgetown University Hospital; Féminas; Dalla Lana School of Public Health, University of Toronto; Universidad de Huánuco; Keck School of Medicine at the University of Southern California; McGovern Medical School at UTHealth; David Geffen School of Medicine, UCLA; David Geffen School of Medicine, UCLA

**Keywords:** Transgender women, HIV prevention, social networks, social support, Peru, Latin America

## Abstract

**Introduction:**

Social networks contribute to normative reinforcement of HIV prevention strategies, knowledge sharing, and social capital, but little research has characterized the social networks of transgender women (TW) in Latin America. We conducted a mixed methods analysis of three network clusters of TW in Lima, Peru, to evaluate network composition, types of support exchanged, and patterns of communication.

**Methods:**

We recruited TW residing in or affiliated with three “*casas trans*” (houses shared among TW) in Lima between April-May 2018. Eligible participants were 18 or older, self-reported HIV-negative, and reported recent intercourse with a cis-male partner. Participants completed demographic questionnaires, social network interviews, and semi-structured interviews to assess egocentric network structures, support exchanged, and communication patterns. Quantitative and qualitative data were analyzed using Stata v14.1 and Atlas.ti, respectively.

**Results:**

Of 20 TW, median age was 26 years and 100% reported involvement in commercial sex work. Respondents identified 161 individuals they interacted with in the past month (alters), of whom 33% were TW and 52% family members. 70% of respondents reported receiving emotional support from family, while 30% received financial support and instrumental support from family. Of the 13 (65%) respondents who nominated someone as a source of HIV prevention support (HPS), the majority (69%) nominated other TW. In a GEE regression analysis adjusted for respondent education and region of birth, being a family member was associated with lower likelihood of providing financial support (aOR 0.21, CI 0.08–0.54), instrumental support (aOR 0.16, CI 0.06–0.39), and HPS (aOR 0.18, CI 0.05–0.64). In qualitative interviews, most respondents identified a cis-female family member as their most trusted and closest network member, but other TW were more often considered sources of day-to-day support, including HPS.

**Conclusion:**

TW have diverse social networks where other TW are key sources of knowledge sharing and support, and family members may also represent important and influential components. Within these complex networks, TW may selectively solicit and provide support from different network alters according to specific contexts and needs. HIV prevention messaging could consider incorporating network-based interventions with TW community input and outreach efforts for supportive family members.

## Introduction

Transgender women (TW) experience disproportionately higher rates of HIV compared to the general population, and face numerous barriers to accessing healthcare and HIV prevention services(1–3). In Peru, where HIV prevalence among TW is more than 30%, HIV prevention interventions must be adapted to address specific barriers faced by TW, leverage existing social networks and support systems, and improve uptake of HIV prevention practices including HIV pre-exposure prophylaxis (PrEP)(4, 5).

Social networks of TW play an important role in shaping HIV prevention attitudes, influencing individual behaviors, contributing to knowledge sharing, and building social capital that may help TW overcome barriers and stigma related to accessing healthcare as well as strengthen advocacy among TW communities(6–11). Recent studies among men who have sex with men (MSM) and TW have acknowledged the role of social networks in influencing HIV prevention behaviors and the potential utility of networks to encourage behavioral change and improve outcomes(12–18). However, different network characteristics, including network size, alter characteristics, individual relationship dynamics, and unique cultural contexts, may differentially influence HIV prevention attitudes, behaviors, and support systems(14, 19, 20).

Very little research has characterized TW social networks in Latin America. Studies of TW networks globally and within Latin America have highlighted the tendency of TW to build social connections with and receive support from other TW(10, 21, 22). Studies in Guatemala and San Salvador have found that TW tended to have smaller, more homogenous networks with higher turnover than MSM(23, 24). More recent research has highlighted potentially more diversity and nuance in TW network composition, support, and communication(12, 22). Most existing network-based HIV prevention interventions for TW are centered around recruitment of TW network members and community leaders to serve as peer navigators(25–27). Studies among both MSM and TW have demonstrated that peer navigation has high acceptability and may facilitate linkage to care, PrEP adherence, and other HIV prevention practices(25–29). However, more research is needed to understand relationship dynamics within networks of TW and the potential roles of other network members, such as family and partners, in providing social support and contributing to social capital. A careful examination of network structures among TW in Latin America is crucial for understanding their potential role in building social capital and promoting HIV prevention.

An improved understanding of the complexities of TW social networks and how various types of support are deployed, solicited, and received within different network relationships can help shape and diversify HIV prevention efforts. We aimed to characterize social network structures of TW living in Lima, Peru and explore patterns of communication and support exchanged within these networks. We conducted a mixed methods study and social network analysis of a group of TW in Lima, Peru, who lived in or were associated with three different “*casas trans*” (houses shared among TW that had significant social overlap) to investigate the formation of these social networks and support structures, and understand the nuanced dynamics of specific relationships within these networks.

## Methods

### Participants and recruitment

Data was collected in May 2018 in Lima, Peru. 20 Participants were recruited from a local TW community organization by a peer navigator. Participants were selected using convenience sampling. Eligibility criteria included: 18 years of age or older, self-identified as a TW, self-reported HIV-negative or unknown HIV serostatus, and reported anal and/or oral sex in the past 12 months.

### Study procedures and data collection

Prior to the initiation of any study procedures, all participants signed an informed consent. Participants completed a demographic questionnaire, a standardized social network interview (SNI), and a semi-structured qualitative interview. Qualitative interviews were conducted by two interviewers fluent in Spanish. One interviewer was a TW from Lima, Peru, and the other was a cis-woman researcher with experience in conducting qualitative interviews about HIV/STIs. The interview guide was developed with feedback from a local TW community organization and was designed to explore individual relationships in participants’ networks, support exchanged, and communication regarding HIV/STIs. The guide was finalized after three pilot interviews. All interviews were conducted in private, audio transcribed, and checked for completeness and accuracy by a member of the research team. Each interview was reviewed promptly to identify preliminary thematic codes. Recruitment ended when thematic saturation was reached. Interviews lasted between 45–60 minutes and participants received a compensation of 40 Nuevos Soles (approximately $13 USD).

### Measures

#### Social network interview.

The SNI asked respondents to identify the individuals with whom they had interacted in the last month (alters) using the following name generator prompt: “Think about the last month (date given), who have you interacted with?”. For each alter, questions assessed alter characteristics such as relationship to respondent (family vs non-family), relationship length, and gender identity (TW vs other gender identity (i.e., cisgender man, cisgender woman)).

Communication modes with each alter were assessed using the following individual questions: “In the last month, who on here have you spent time with face to face?”; “In the last month who on here have you connected with using your phone?”; and “In the last month who on here have you connected with using a computer?” (dichotomized to yes/no for each mode). Communication frequency was assessed with: “Which of these people do you communicate with at least once per week?” (dichotomized to weekly vs. non-weekly). Respondents indicated their three “closest” network members from their list, to allow further elaboration during subsequent qualitative interviews.

Social support was assessed and aggregated to create measures of different types of support: emotional, financial, HIV prevention, and instrumental support. Emotional support was assessed with the following questions: “Who makes you feel liked or loved?”; “Who makes you feel respected or admired?”; “Who can you confide in?”; “Who agrees with or supports your actions or thoughts?”. Financial support was assessed with: “If you needed to borrow $10 or some other immediate help, who could help you with this?”. HIV prevention support (HPS) was assessed with: “Does anyone remind you to take your medicine to prevent HIV?”; “Has anyone taken you to the doctor or other HIV-related appointments?”; “To the best of your knowledge, who is HIV positive?”. Instrumental support was assessed with: “If you were confined to a bed for several weeks, who could help you?”; “If you needed assistance, who would help you with housework?”. If an alter was nominated for at least one of the emotional support, HPS, financial support, or instrumental support questions, that alter was considered to provide that support. All four social support variables were dichotomized (yes/no to providing each type of support).

### Data analysis

We sought to describe the composition of respondents’ social networks, types of support exchanged, directionality of support exchanged, and communication patterns with alters. We used a mixed methods approach to describe respondents’ networks and investigate the types of support provided, while exploring the nuances of these networks. We aimed to understand the contexts in which these networks form and assess respondents’ views and experiences within their networks.

#### Quantitative.

Descriptive statistics including mean, median, range, and frequency distributions were used to describe respondent and alter characteristics. Associations between outcomes (types of support and communication patterns) and alter characteristics were evaluated using regression analysis with generalized estimating equations (GEE) with a binomial distribution and exchangeable correlation matrix to account for within-participant repeated measures.

Our outcomes of interest were: 1) Social network support, measured by perceived emotional, HPS, financial, and instrumental support from alters; and 2) Communication with alters (i.e., communication mode and frequency). GEEs were used to calculate unadjusted and adjusted odds ratios (OR) of reported support and communication types by 1) relationship type and 2) gender identity. Both analyses were adjusted for respondent education level and region of birth. Quantitative data were analyzed using Stata.

#### Network graphs.

Social network graphs were created in R using the igraph package to represent ties between respondents and alters. Graphs were created to highlight the provision of each type of support (emotional, instrumental, financial, and HPS) by alters. Alter characteristics represented in network graphs include gender identity (TW, cisgender man, cisgender woman) and relationship to respondent (relative, friend, current or former partner, and health promoter).

#### Qualitative.

Data from semi-structured interviews was analyzed in Atlas.ti using an immersion crystallization approach. This technique allows researchers to immerse themselves in the data and reflect on themes that arise, or crystallize, during this process(30). An a priori codebook was developed using a deductive approach based on literature review and themes outlined in the interview guide. Two researchers read through interview transcripts to identify overarching themes and determine when thematic saturation had been reached. The a priori codebook was then used by one researcher to code the transcripts line-by-line. Additional codes were added and codes were merged as needed during this process.

## Results

### Respondent demographics

Median age of respondents was 26 years (IQR 21.5–32.5), and all reported current engagement in commercial sex work at the time of data collection (Table 1). 85% of respondents reported living in one of the three “casas trans”. Five respondents (25%) were born in Lima/Callao. Among respondents born elsewhere, median time spent living in Lima was 5 years (IQR 1.6–7). 35% of respondents reported being in a partnership and 55% reported having one or more dependents.

### Network composition and alter characteristics

In SNIs, respondents nominated a total of 161 alters they had interacted with in the past month (Table 2). Median age of alters was 26.5 years (IQR 22–39.5). Median social network size was 7 (IQR 6–10.5, range 3–17). 33% of alters were cisgender males, 34% were cisgender females, and 33% were TW. All respondents nominated at least one family member as part of their network, with family comprising 52% of alters. Seven respondents reported being in a partnership at the time of the study.

### Regression analysis

There was no difference in the perceived provision of emotional support based on alter relationship type (Table 3) or gender identity (Table 4). Family members were less likely to provide financial support (adjusted (a)OR 0.21, CI 0.08–0.54) compared to non-family members. TW alters were more likely to be considered sources of financial support (aOR 3.08, CI 1.22–7.75) compared to alters who were not TW. Being a family member was associated with a lower likelihood of providing instrumental support compared to non-family member alters, while being a TW was associated with a higher likelihood of providing instrumental support (aOR 6.24, CI 2.81–13.84) compared to non-TW alters. Family members were less likely to provide HPS than non-family alters, while TW alters were more likely to provide HPS (aOR 3.24, CI 1.18–8.92) compared to non-TW alters.

Respondents reported weekly communication with 77% of non-family and 45% of family alters. Respondents reported weekly communication with 83% of TW alters compared to 49% of non-TW alters (aOR 6.95, CI 2.82–17.10). Family members were significantly more likely to communicate with respondents via telephone and computer and less likely to communicate in person. In comparison, alters that were TW were more likely to communicate with respondents in person and less likely to communicate via phone.

### Social networks represented important and nuanced sources of different types of support

#### Emotional/social support.

**Figure 1:**
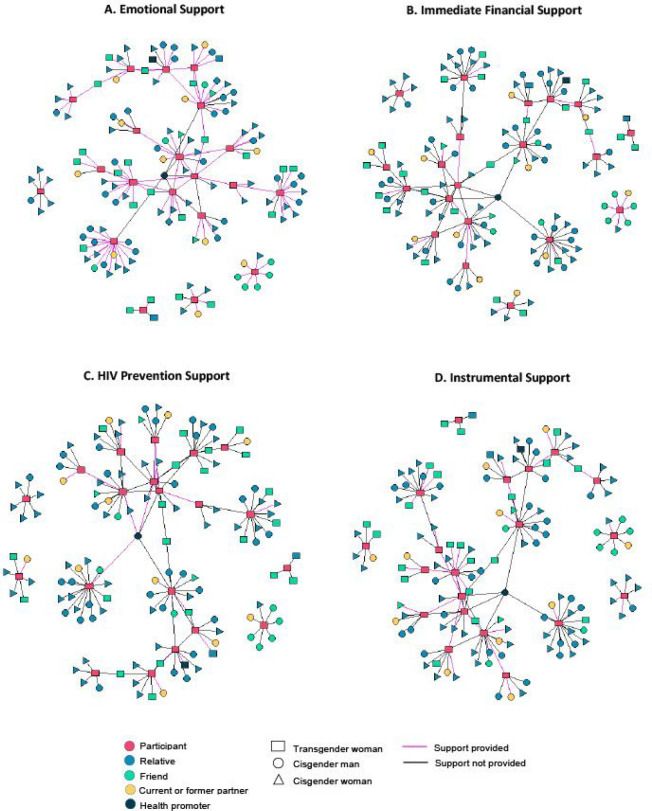
**A-D** Social network graphs depicting alters nominated as potential sources of emotional support, instrumental support, financial support, and HIV prevention support.

In SNIs, 75% of respondents reported receiving emotional support from family members (Fig. 1A). In qualitative interviews, almost all respondents identified a family member, most often a cisgender female such as a mother or sister, as one of the most trusted, influential, and closest members of their network. Families were widely considered an important source of social and emotional support, which was often simply associated with their role as family members. Several respondents echoed the sentiment that they trusted and felt closest to their mothers “because she is my *mamá*.”

A few respondents described family members who did not accept their gender identity and with whom they communicated infrequently, but sometimes still considered “close” network members. However, when families accepted respondents’ identities and openly discussed issues including HIV/STI risk, gender-affirming procedures, and/or sex work, respondents often described feeling encouraged to engage in HIV preventive behaviors and empowered in the face of discrimination.

“I think she [mother] felt bad when I started to get depressed because I was feeling the bullying from people, so we talked more and she understood my suffering and my desire to be a woman physically because internally I have always been one. So then she said, ‘I’m going to support you.’” – *38 years old, from Lima*

“ She [sister] always tells me ‘whatever you decide I will always support you, I have no reason to be judging you, nor telling you things’.” – *22 years old, from Cajamarca*

Of the eleven respondents (55%) who identified a current or former romantic partner as part of their network, ten reported receiving emotional support from this partner. In interviews, several respondents described partners who made them feel respected and confident, which contributed to empowerment against stigma and discrimination.

“He makes me have a lot of confidence in myself. With the other partners I had, it was like they hid me, ‘don’t come to my work, wait for me on the corner.’ Him no, ‘sit at my side while I work’ or ‘come to my house, come to the room where I live’. Things like that.” – *38 years old, from Lima*

In SNIs, 70% of respondents reported receiving emotional support from other TW. In qualitative interviews, respondents elaborated on the unique and important role that other TW had in creating new social structures and channels of support within their community. Many respondents reported moving from other regions to come to Lima and feeling supported by TW in the area who welcomed and oriented them to the local community.

“Everyone [in my family] lives in Pucallpa… All I have here are the trans girls that are also my friends.” – *28 years old, from Pucallpa*

Several respondents who did not have supportive family members cited this fact as another key reason for moving to Lima, where they found a more supportive environment. These new and alternative systems of support created by TW for other TW contrasted significantly with the more traditional family and romantic relationship structures respondents described. Support systems within the trans community were generated organically because of shared identities and experiences, and were crucial for defining social norms, sharing knowledge, and facilitating labor opportunities.

“I decided to change physically [transition], so I made the decision to come [to Lima] and I contacted the girls here and at that time they were already working in this environment [sex work].” – *28 years old, from Pucallpa*

“I simply came [to Lima] because I had friendships here, and then I stayed. My friend encouraged me, ‘don’t go, get to work here, here we’ll make money’… and I stayed to work.” – *19 years old, from Tarapoto*

#### Financial support.

The source and directionality of financial support in these networks varied based on the type of financial support and respondents’ relationship with specific network members. Eighteen respondents (90%) reported having a potential source of financial assistance (Fig. 1B). Of those respondents, only six (33%) nominated a family member. In qualitative interviews, respondents elaborated on patterns of more consistent financial support. Notably, almost half of respondents reported having no sources of consistent financial support. Several others reported that they were responsible for providing financial support to their families.

“My family is depending on me, they’re passing through a [difficult economic] situation. I send them 100, 150, 200 soles weekly.” – *28 years old, from Pucallpa*

“I support [my mother] economically… I send her money weekly for her food costs, or for my niece’s costs, for her school.” – *27 years old, from Piura*

Most respondents with long-term partners reported being financially supported by these partners in some way, such as helping with rent payments. With other TW, financial support was often described to be bidirectional; TW helped each other out as needed.

“When sometimes she doesn’t have [money] and I do, and sometimes when I don’t have [money] and she does, I invite her to eat like this… and she does the same. We support each other.” – *19 years old, from Tarapoto*

Directionality of financial support varied with each relationship type. With families, financial support tended to be passed from respondents to their families; with primary partnerships, support came almost exclusively from partners; and between respondents and other TW in their networks, financial support was exchanged both ways.

#### Instrumental support.

Participants nominated friends, especially other TW, more often as potential sources of instrumental support. Of the 19 respondents who identified any source of instrumental support, six (32%) nominated a family member and 15 (79%) nominated at least one other TW (Fig. 1C). The bidirectionality of this support among TW was highlighted in qualitative interviews.

“When I need to eat, she [TW friend] supports me because she has a kitchen, she cooks or she lends it to me, and also she lends me clothes, or I also lend her things and it’s like this.” – *23 years old, from Pucallpa*

#### Health and HPS.

HPS was less prevalent in respondents’ networks. Of the 13 respondents (65%) who nominated someone as a source of HPS, a majority (69%) nominated other TW (Fig. 1D). Qualitative interviews shed light on the ways in which HPS was exchanged among TW, as well as the potential role of some family members in supporting HIV prevention and other health behaviors.

Respondents who reported receiving health-related support from family or partners primarily described support in the form of periodically checking on respondents and providing general encouragement to maintain a healthy lifestyle, often avoiding explicit discussions about sexual health, HIV/STI prevention, and trans-specific issues such as access to gender-affirming healthcare. Family members’ expressions of concern and questions about wellbeing were not always well-articulated and were instead encompassed by the general advice to “take care”.

“She [mother] is always giving me advice, telling me to take care of myself, how am I, she is always asking, if I have eaten lunch or not” – *23 years old, from Pucallpa*

“We just chat like this ‘how are you girl, are you doing well?’ My brothers, my sisters-in-law, everyone, ‘Take care of yourself… are you doing well?’” – *36 years old, from Pucallpa*

Several respondents reported having supportive family members with whom they could openly discuss issues related to HIV/STIs. Though less common, some respondents described receiving encouragement from family to engage in HIV/STI prevention, with one respondent describing that her mother reminded her to take PrEP and another stating that her mother buys her condoms.

“When [my mother] found out I was gay…she accepted me for who I am. [She told me] to take care, that there are [sexually transmitted] diseases, that I should always use protection.” – *19 years old, from Pucallpa*

More often, when respondents felt comfortable discussing HIV/STI prevention with family members, respondents were the ones initiating and leading the conversation to educate their family about these issues.

“I have explained to [my mother] the risks that I’m exposed to from working in the street… I tell her that there are various sexually transmitted diseases like HIV, AIDS, syphilis.” – *27 years old, from Piura*

Some even used their experience and knowledge to advise younger family members, such as siblings, nieces, and nephews, about HIV/STI prevention.

“I told [my sister], ‘you have to use a condom so that you don’t get pregnant, another reason is there are plenty of sexually transmitted infections like HIV, the condom isn’t just for pregnancy, but also for other risks that you need to protect yourself from,’ I told her.” – *23 years old, from Pucallpa*

A similar pattern existed in relationships with romantic partners. Most partnered respondents described partners checking in on their health generally or even taking care of them when they were sick. However, with regard to HIV/STI prevention, respondents felt responsible to educate and provide that support to their partners.

“He [partner] did know about HIV but didn’t know some things that, I from experience, know a ton. About precautions…about how to avoid contracting [HIV].” – *38 years old, from Lima*

In contrast, when describing their TW networks, respondents reported that other TW provided more active HPS in the form of knowledge sharing, facilitating access to services, and encouraging HIV preventive behaviors, especially in the context of sex work. Often, older or more experienced TW educated younger or newly arrived TW, such as those that were new to the city and/or the profession of sex work. Several respondents reported that the friend that introduced them to sex work and this community of TW also taught them about prevention.

“When I started working in this [sex work]… a trans friend [told me] that I always have to use condoms, always in this routine that I have, I have to use them… because it is sex work.” – *31 years old, from Trujillo*

In general, the directionality of HPS varied similarly based on relationship type. Though some respondents received HPS from family, TW were typically the source of HPS and education for their non-TW network members. In relationships with other TW, the provision of this type of support was often bidirectional.

## Discussion

In this mixed methods analysis of 20 TW living in Lima, Peru, we found that respondents had diverse social networks comprised of family members, partners, and friends, particularly other TW. Respondents’ networks represented key sources of emotional, financial, instrumental, and HPS, with clear differences seen in the kind of support received or provided according to relationship type. Most respondents’ networks could be defined by a central division between TW networks and family networks, with little overlap between the two. Respondents received the most support from other TW in their networks, though family and partners were also considered important network members, and respondents selectively sought out and provided support within these networks based on specific situations and contexts.

Family members and TW friends represented the majority of respondents’ social networks. Notably, respondents’ networks of other TW were interconnected, with several respondents nominating one another or the same TW friends. However, no overlap was observed between family networks and TW networks. This observation suggests that respondents are part of at least two distinct communities or micro-networks, receiving different types of support from each and likely navigating different norms, expectations, and interactions with each. This concept was reinforced in qualitative interviews where both TW networks and family networks represented crucial sources of support for respondents, but the type of support exchanged, the directionality of this support, and the contexts in which it was sought out differed based on these distinct network relationships.

Almost all respondents nominated family members as part of their network and as sources of several different types of support. Prior studies have shown that TW networks tend to be smaller compared to MSM networks and may be comprised primarily of other TW, but very little research in Latin America has explored the role of family members in the networks of TW(23, 24). A Guatemala City study found that TW had small networks where few listed family as part of their network and all expressed that their gender identity negatively impacted relationships with family(23). Family members’ presence in TW networks is understudied in Latin America and regional differences and unique cultural contexts likely impact these relationships. Though themes of family rejection of gender identity emerged in our analysis(23, 31), most respondents described a high degree of closeness to family members. Participants tended to place significant value on family and the emotional support family members provided, even if they did not provide other types of support. This sentiment may represent attitudinal manifestations of *familismo* (familism) in Latin American cultures, in which higher value is placed on the family unit, causing members of the same family to share substantial in-group feelings and a sense of obligation to provide emotional and instrumental support to their families(32). *Familismo* in Latin America has been shown to improve mental health outcomes in several studies(32), though this has not been studied in populations of TW. Our study contributes to existing literature by highlighting the unique and nuanced relationships that respondents had with family members in their network and the previously unexplored role of family members in providing HPS, even if they are not the primary source of this support.

Though our results suggest that TW in Peru may be substantially close to their families, their social networks are also defined by unique contexts introduced by the prevalence of internal migration from provinces to Lima. Most respondents who moved to Lima relied less on familial support after migration, instead forming new social structures within the local TW community. This is further demonstrated by the differing communication patterns between respondents and family compared to other TW. Respondents were less likely to communicate with family members face-to-face and were more likely to communicate with TW alters face-to-face and at least weekly. These results might be explained by most respondents’ lack of physical proximity to family as well as the relative importance of new social structures formed by TW living in Lima based on shared identities, experiences, and routines. This type of internal migration and TW community formation has been documented in the literature(35, 36). The impact of this migration on the construction of new social structures is reflected in the concomitant development of new social support systems among TW within these distinct socio-geographic contexts.

TW alters had a higher likelihood of being considered a potential source of instrumental, financial, and HPS compared to non-TW alters. This support was often described as bidirectional between respondents and other TW in their network. Our results contribute to existing literature describing the social support provided by TW networks and provide additional detail about specific types of support and directionality of support that ultimately contributes to the formation of unique social structures and generation of social capital in these networks(12, 37). Our results suggest a cycle of knowledge sharing and support exchange in which more experienced TW helped to educate and integrate younger TW or those new to the community who then went on to provide various types of support to their other network members, including other TW, families, and partners. This highlights the role of TW as leaders within their networks and origin points for the spread of knowledge within communities, especially regarding HIV prevention. This observation also demonstrates the alternative social systems that TW may create within their families or communities because of social marginalization and exclusion from many traditional social and community structures. These alternative systems can provide the basis for delivery of HIV prevention and treatment resources and empowerment of TW communities.

Another important network relationship highlighted in our study was that between respondents and their primary partners, with over half of respondents reporting a current or former romantic partner in their network. Relationships between TW and their primary partners are largely undefined in the current literature. Recent studies have investigated cisgender men who have sex with transgender women (MSTW) as distinct from MSM, but very few studies have explored the nature of the relationships between TW and the partners they consider primary partners(38–42). Our study suggests that primary partners may represent an additional source of social and financial support for TW, and that significant discussion surrounding HIV/STIs occurs in these relationships. However, it should be noted that many TW may have other sexual partners in addition to a primary partner(23, 43, 44). Non-primary sexual partners were not represented in respondents’ network graphs and may not represent a significant source of support for TW but are a potentially important source of HIV/STI acquisition risk. More research is needed to better characterize TW partnership contexts, including both “primary” and other sexual partners (e.g., casual partners, anonymous contacts, and sex work clients), to better understand this complex network of contacts that may influence HIV/STI transmission and to consider ways to engage these partners in HIV prevention interventions or link them to care.

## Limitations

Several limitations of this study should be noted. Our social network analysis focused on egocentric networks, which relies on respondents to provide information about their networks and individual alters. This process can be subject to inaccuracy, a limitation which we tried to mitigate using name generator prompts that included many possible relationship categories to facilitate recall. In addition, while other studies have found that the social networks of TW may be transient(23, 24), our cross-sectional analysis was not designed to evaluate this possible characteristic. A limited number of questions were used to define HPS in the SNI. For example, in qualitative interviews we found that encouraging condom use and facilitating access to condoms may represent a way that network members support respondents’ HIV prevention, but this was not reflected in the SNI questions. This study is also limited to TW in Lima, Peru, and may not be generalizable to other contexts. Finally, the regression analysis used in this study may be limited due to small sample size.

## Conclusions

This study sheds light on social network composition and support structures among a group of TW in Lima, Peru. Though limited to a single community linked to three *casas trans*, these findings contribute to the existing literature regarding TW social networks in Latin America and highlight several implications for future social network research and interventions. TW have complex networks of support that span families, romantic partners, friends and acquaintances. These networks are primarily divided based on trans identity, with extensive overlap existing among TW network members, but not among family or partners. Types of perceived support varies significantly according to relationship type. The diversity of these networks and existence of two distinct micro-networks – one of primarily TW and one of family – may facilitate the exchange of several different types of support, all of which are valued by respondents. This division and selective utilization of each micro-network has important implications for HIV prevention interventions and should be further studied. Primary partners were also frequently nominated as alters who provided varying amounts of support, but more research is required to further characterize this group. Understanding the complex structures within these networks is essential to their potential use for the delivery of HIV prevention and treatment interventions.

## Figures and Tables

**Table 1:** Respondent demographics

Characteristics	Respondents (N = 20)
	n (%)
Age, median (IQR^1^)	26 (21.5–32.5)
Region of birth	
Lima/Callao	5 (25)
Provinces	15 (75)
Northern coastal	5 (25)
Amazon	9 (55)
Andes	1 (5)
Years in Lima^2^, median (IQR)	5 (1.6–7)
Currently living in trans house	17 (85)
Engagement in sex work	20 (100)
Education	
Did not complete secondary school	9 (45)
Completed secondary school or greater	11 (55)
Household income^3^	
300–500 Soles/month	2 (10.5)
501–1500 Soles/month	16 (84.2)
1501–3000 Soles/month	1 (5.3)
Number of dependents, median (range)	2 (0–8)
Relationship status	
Single	13 (65)
In a partnership	7 (35)

1 Interquartile range

2 If born in other region (n = 15)

3 N=19 due to missing data

**Table 2:** Alter characteristics

	Alters (n = 161)
	n (%)
Gender identity (n = 159)^1^	
Cisgender male	53 (33)
Cisgender female	54 (34)
Transgender woman	52 (33)
Age in years, median (IQR)	26.5 (22–39.5)
Relationship to respondent	
Parent	21 (13)
Sibling	42 (26)
Other family member	21 (13)
Friend	58 (36)
Current or former romantic partner	11 (7)
Health promoter	6 (4)
Landlord	2 (1)
Relationship length in years^2^, median (IQR)	4 (1.8–7)

1 N=159 due to missing data

2 If not family member (n = 77)

**Table 3:** Support and communication patterns in respondent social networks by alter relationship to respondent

	Network members n (%)		Unadjusted analysis	Adjusted analysis^1^
	Family (n = 84)	Not family (n = 77)	Odds ratio (95% CI)	Odds ratio (95% CI)
Communication patterns				
Telephone	41 (49)	11 (14)	5.73* (2.69–12.17)	6.19* (2.80–13.70)
In person	13 (15)	56 (73)	0.07* (0.03–0.15)	0.07* (0.03–0.15)
Computer/social media	18 (21)	6 (8)	3.13* (1.19–8.29)	3.12* (1.18–8.25)
Communicate weekly (any mode)	38 (45)	59 (77)	0.28* (0.14–0.54)	0.25* (0.13–0.51)
Emotional support	61 (73)	59 (77)	0.77 (0.39–1.55)	0.77 (0.38–1.56)
Immediate financial support	7 (8)	23 (30)	0.23* (0.10–0.58)	0.21* (0.08–0.54)
HIV prevention support	3 (4)	13 (17)	0.19* (0.05–0.65)	0.18* (0.05–0.64)
Instrumental support	7 (8)	28 (36)	0.16* (0.07–0.39)	0.16* (0.06–0.39)

1 Adjusted for respondent education level and region of birth

* Statistically significant: 95% CI does not cross 1

**Table 4:** Support and communication patterns in respondent social networks by alter gender identity

	Network members^1^ n (%)		Unadjusted analysis	Adjusted analysis^2^
	Transgender woman (n = 52)	Other gender identity (n = 107)	Odds ratio (95% CI)	Odds ratio (95% CI)
Communication patterns				
Telephone	4 (8)	46 (43)	0.12* (0.04–0.35)	0.12* (0.04–0.36)
In person	41 (79)	28 (26)	10.78* (4.86–23.92)	13.5* (5.71–32.11)
Computer/social media	5 (10)	19 (18)	0.49 (0.17–1.37)	0.51 (0.18–1.48)
Communicate weekly (any mode)	43 (83)	52 (49)	5.13* (2.25–11.71)	6.95* (2.82–17.10)
Emotional support	42 (81)	76 (71)	1.82 (0.83–4.00)	1.77 (0.79–3.98)
Immediate financial support	13 (25)	16 (15)	2.23 (1.00–4.99)	3.08* (1.22–7.75)
HIV prevention support	8 (15)	8 (7)	2.24 (0.82–6.14)	3.24* (1.18–8.92)
Instrumental support	22 (42)	13 (12)	5.73* (2.65–12.39)	6.24* (2.81–13.84)

1 N=159, gender identity data is missing for 2 nominated alters

2 Adjusted for respondent education level and region of birth

* Statistically significant: 95% CI does not cross 1

## Data Availability

Data used for this study are available from the corresponding author on reasonable request.

## Abbreviations

TW: transgender women
HIV: Human immunodeficiency virus
GEE: generalized estimating equation
OR/aOR: odds ratio/ adjusted odds ratio
HPS: HIV prevention support
PrEP: pre-exposure prophylaxis
MSM: men who have sex with men
STI: sexually transmitted infection
SNI: social network interview
MSTW: men who have sex with transgender women
